# Role of Berry Bioactive Compounds on Lipids and Lipoproteins in Diabetes and Metabolic Syndrome

**DOI:** 10.3390/nu11091983

**Published:** 2019-08-22

**Authors:** Arpita Basu

**Affiliations:** Department of Kinesiology and Nutrition Sciences, School of Integrated Health Sciences, 4505 S Maryland Parkway, University of Nevada Las Vegas, Las Vegas, NV 89154, USA; Arpita.basu@unlv.edu; Tel.: +01-702-895-4576

**Keywords:** Lipids, Lipoproteins, cranberry, blueberry, strawberry, LDL, metabolic syndrome

## Abstract

Blood lipids are an important biomarker of cardiovascular health and disease. Among the lipid biomarkers that have been widely used to monitor and predict cardiovascular diseases (CVD), elevated LDL and low HDL cholesterol (C), as well as elevated triglyceride-rich lipoproteins, deserve special attention in their predictive abilities, and thus have been the targets of several therapeutic and dietary approaches to improving lipid profiles. Among natural foods and nutraceuticals, dietary berries are a rich source of nutrients, fiber, and various types of phytochemicals. Berries as whole fruits, juices, and purified extracts have been shown to lower total and LDL-C, and increase HDL-C in clinical studies in participants with elevated blood lipids, type 2 diabetes or metabolic syndrome. This short review aimed to further discuss the mechanisms and magnitude of the lipid-lowering effects of dietary berries, with emphasis on reported clinical studies. Based on the emerging evidence, colorful berry fruits may thus be included in a healthy diet for the prevention and management of CVD.

## 1. Lipids and Cardiovascular Health

Cardiovascular disease (CVD) is the leading cause of global mortality and a growing worldwide public health problem. Among the multiple traditional risk factors of CVD, blood lipid level, especially blood cholesterol level, is an established predictor of CVD risks and subsequent complications. Low-density lipoprotein cholesterol (LDL-C) is a well-established risk factor for CVD, and was recognized by the National Heart, Lung, and Blood Institute in formulating the National Cholesterol Education Program (NCEP) more than 30 years ago to educate both the medical community and the public about the need to lower levels of blood cholesterol in order to reduce the risk of major vascular events (NCEP 1998) [1]. Based on the guidelines of the American College of Cardiology and the American Heart Association, the recommended range of serum LDL-C is 70–100 mg/dL, total cholesterol (TC) less than 200 mg/dL, and triglyceride ≤ 150 mg/dL for the primary prevention of CVD [2]. In a meta-regression analysis of 49 clinical trials with 312,175 participants, each 1 mmol/L reduction in LDL-C level was associated with a 23% risk reduction of major vascular events for statins, and a 25% risk reduction for non-statin interventions, including dietary approaches to lower LDL-C [3] Studies have further revealed that residual cardiovascular risk remains after LDL-C goals are achieved with lipid-lowering treatments, especially in high-risk patients such as those with type 2 diabetes or metabolic syndrome. This residual risk can be attributed to the low high-density lipoprotein (HDL) and high triglyceride-rich lipoprotein levels routinely measured in clinical care. Thus, in addition to LDL-C, triglyceride, HDL-C, non-HDL-C, total apolipoprotein (apo) B, apoB/A-I, and TC/HDL-C levels are considered significant predictors of cardiovascular events and have been validated in large prospective studies in cardiovascular and diabetes epidemiology [4,5,6]. Further, qualitative changes in lipids, such as shifts among low, medium, and large lipid particle size and molar concentrations have been associated with CVD risks and events beyond conventional lipid profiles [7,8]. Thus, lipid and lipoprotein subclasses based on size and density have been widely examined in lipid-lowering interventions. 

Dietary and food-based approaches for lowering lipids have been widely practiced as a secondary or adjunct strategy, especially in achieving weight loss and healthy lifestyle goals associated with favorable lipid profiles. Among these dietary approaches, several foods and supplements containing phytosterols, fiber, and polyphenols have gained recognition for their lipid-lowering effects. In this context, berry fruits and their products have shown much promise in offering several cardiovascular benefits, including those related to favorable lipid profiles [9,10]. In a meta-analysis of clinical studies on *Vaccinium* berries, significant lipid-lowering effects were observed based on pooled analysis of 16 clinical studies in 1109 participants [11]. This communication aimed to provide further insights into the role of dietary berries in blood lipid management, with special emphasis on the reported clinical studies. 

## 2. Review Methods

We herein have focused on clinical studies that involved the administration of whole berries, juices, and their extracts as the study agent(s). We conducted a search using PUBMED and the clinical trial registry (ClinicalTrials.gov) for studies reported in 1999–2019 using key words such as “berries”, “berry extracts”, “serum cholesterol”, “LDL cholesterol”, “HDL cholesterol”, “LDL particle”, “apolipoprotein B”, and “C-reactive protein”. The search inclusion criteria included terms as follows: blueberry, cranberry, strawberry, berry extracts, metabolic syndrome, type 2 diabetes, serum cholesterol, lipoproteins, particle size, inflammation, cohort, and clinical trial; exclusion criteria included terms as follows: children, rats, cell model, polyphenol extracts, fruits, vegetables, myocardial infarction, atherosclerosis. This search aimed to keep the focus of our communication on the role of whole berries and their bioactive compounds in the management of lipids and lipoprotein profiles and biomarkers of inflammation in adults with diabetes and metabolic syndrome.

### 2.1. Berries: Composition and Nutritional Value

Berries have a wide range of nutrients and bioactive compounds that have been extensively reviewed for their protective effects against CVD [12]. The commonly consumed berries in the US include blackberry, black raspberry, blueberry, cranberry, red raspberry, and strawberries. Less commonly consumed berries include acai, blackcurrant, chokeberry, and mulberries. In general, berries are low in calories and are high in moisture and fiber, and contain natural antioxidants such as vitamins C and E, and micronutrients such as folic acid, calcium, selenium, alpha and beta carotene, and lutein. For example, 100 g frozen unsweetened strawberries provides only 35 kcal, and thus can be included as a low-calorie, fat-free, nutrient-dense snack in the dietary management of CVD [13]. Berries, including the commonly consumed blueberries and strawberries, are naturally rich in polyphenolic flavonoids, with high proportions of flavonoids including anthocyanins and ellagitannins. Anthocyanins comprise the largest group of natural, water-soluble, plant pigments and impart the bright colors to berry fruits. Approximately 400 individual anthocyanins have been determined, and these are generally more concentrated in the skins of fruits, especially berry fruits. However, red berry fruits, such as strawberries and cherries, also have anthocyanins in their flesh. Studies suggest that Americans consume an average of 12.5–215 mg of anthocyanins per day [14].

### 2.2. Berries and Lipids: Animal Models

Animal models provide mechanistic insights into the role of berries in lipid and lipoprotein metabolism. Berries and their bioactive constituents, such as polyphenolic flavonoids and phenolic acids, have been shown to increase paraoxonase (PON) activity associated with the antioxidant function of HDL cholesterol, and also increase the hepatic synthesis of apolipoprotein A-I. For example, acai berry, native to the Amazon region, has been reported to increase serum activity of PON1 and levels of HDL cholesterol, and increase fecal cholesterol excretion in rat models of hyperlipidemia and hepatic steatosis [15,16,17]. Commonly consumed dietary berries, such as blueberries and strawberry extracts, have also been demonstrated to improve lipid profiles by downregulating the activity of genes related to fatty acid synthesis, causing regression of aortic lesions, as well as decreasing inflammation and oxidative damage in these animals [18,19]. Strawberry and blueberry pomace, a byproduct from industrial fruit processing, has emerged as a promising source of antioxidants and micronutrients, and has been tested for its health benefits by many researchers, mainly as an alternative and cost-effective way of supplementing the human diet with berry polyphenols. In two separate studies, blueberry and strawberry pomace supplementation improved multiple metabolic parameters, including plasma and liver cholesterol, insulin resistance, and abdominal fat content in fructose-fed animals [20,21]. As expected, the polyphenol content of the berry pomace was shown to play an important role in its lipid-lowering effects, based on larger decreases in liver cholesterol content in the high polyphenol pomace group when compared to the regular-pomace-fed animals [20]. Thus, these studies have provided evidence for the protective action of polyphenols and fiber mediated by berries in rat models of hyperlipidemia, and further studies are needed to assess the possible role of these compounds in the prevention of metabolic and lipid disorders in humans. 

### 2.3. Berries and Lipids: Epidemiological Studies

More than a decade ago, Djousse et al. reported a large epidemiological study on the inverse association of fruit and vegetable intake with serum LDL cholesterol concentration in participants from the National Heart, Lung, and Blood Institute (NHLBI) Family Heart Study [22]. Since then, accumulating epidemiological evidence has further revealed the inverse association of dietary berries with chronic disease outcomes linked to dyslipidemia and hyperlipidemia, especially insulin resistance, type 2 diabetes, coronary artery disease, and non-fatal myocardial infarction (MI) in large prospective studies of adult men and women [23,24,25]. Among the bioactive compounds in berries, anthocyanins responsible for the red/blue hue in these fruits have been mainly linked to their protective effects. These compounds have been shown to directly improve dyslipidemia and lipoprotein profiles, as well as to decrease surrogate markers of atherosclerosis in reported clinical trials [26,27]. In one of the epidemiological studies, food-based analyses revealed a trend toward a reduction in risk of MI with increasing intake of the two main sources of anthocyanins from strawberries and blueberries, with a 34% decrease in risk for those who consumed >3 portions per week compared to those who consumed these berries less than once a month. These data are important from a public health perspective because these fruits can be readily incorporated into the habitual diet [25]. In another longitudinal study, higher intakes of red/purple fruits and vegetables were associated with significantly lower serum total cholesterol in adults [28]. In the Women’s Health study, strawberry intake was specifically associated with marginally significant but nonetheless lower levels of C-reactive protein, a stable marker of inflammation which has been shown to be elevated with higher serum lipids [29]. Of practical relevance was that these significant associations were observed at intakes as low as two servings/week of strawberry intake in women. Thus, on the basis of this epidemiological evidence and related mechanistic insights, adding red and purple berries may be considered a prudent dietary choice in combating the lipid abnormalities that explain many of the underlying causes of cardiovascular complications in adults. 

### 2.4. Berries and Lipids: Clinical Studies

As summarized in Table 1 and Table 2, dietary berries as whole fruits and in different processed forms have been shown to decrease circulating levels of conventional lipids, as well as shifting lipid and lipoprotein subclasses to a less atherogenic profile in adults with one or more cardiovascular risks. In most of these studies, berry intervention was consistently shown to decrease total and LDL cholesterol [13,26,27,30,31,32,33,34] and increase HDL cholesterol [26,27,33,35], thereby lowering cardiometabolic risks in these adults. While the lipid-lowering effects of whole berries may be supported by several bioactive compounds in the fruit, including their fiber and phytosterol content, studies using commercially available berry juice products, especially low calorie cranberry juice [35,36] have also shown similar beneficial effects of lowering triglycerides and increasing HDL cholesterol. These clinical findings have been supported by epidemiological data from the NHANES 2005-2008 survey, reporting consumers of higher proportions of cranberry beverage (approximately 221 mL/day) were predicted to be normal weight (BMI < 25 kg/m^2^) with lower waist circumference, and had significantly lower triglycerides and CRP than non-consumers [37]. These data provide evidence for the hypolipidemic and anti-atherosclerotic actions of berry fruits and juices in humans. 

In addition to measuring lipid outcomes related to conventional lipids, findings reported by Qin et al. [26] and Zhu et al. [11] provide further mechanistic insights on the hypolipidemic effects of purified berry extracts in adults with independent cardiovascular risks such as dyslipidemia/hyperlipidemia and type 2 diabetes. In a 12 week randomized placebo-controlled trial in adults with dyslipidemia, purified anthocyanins derived from bilberries and black currants were shown to decrease the mass and activity of cholesteryl ester transfer protein (CETP) [26]. CETP is a plasma protein that mediates the removal of cholesteryl esters from HDL in exchange for a triglyceride molecule derived primarily from either LDL, VLDL, or chylomicrons. Thus, CETP inhibition has been shown to be a possible mechanism for the elevation of HDL cholesterol and decrease of LDL cholesterol [38]. In another study in participants with type 2 diabetes, supplementation of a similar berry anthocyanin extract was shown to decrease specific plasma apolipoproteins, especially apolipoprotein B and CIII, that have been associated with increased risks of atherosclerotic CVD in epidemiological observations [11,39,40]. 

Table 3 highlights clinical studies examining the role of dietary berries in decreasing biomarkers of inflammation related to CVD and atherosclerosis. Our group has previously reported the role of blueberries, strawberries, and low-calorie cranberry juice in lowering biomarkers of oxidative stress and adhesion molecules following supplementation in free-living adults with metabolic syndrome [12,43,44]. However, few studies using berry supplementation have shown an effect on key biomarkers of inflammation, such as C-reactive protein (CRP) and interleukin-6 (IL-6), associated with increased CVD risks. Inflammation, demonstrated primarily by elevated levels of serum CRP, has been associated with insulin resistance and metabolic syndrome [45]. Adipose tissue also secretes adiponectin, a protein showing anti-inflammatory activity, which inhibits tumor necrosis factor-α, adhesion molecule expression, and nuclear transcriptional factor kB signaling, a pivotal pathway in inflammatory reactions in endothelial cells, and in the propagation of atherosclerosis [46,47]. In a few reported studies, cranberry juice has been shown to increase levels of circulating adiponectin, and decrease CRP in adults with features of metabolic syndrome [36,48], while biomarkers of inflammation were not altered in other reported studies, as shown in Table 3. Thus, based on the known differences in polyphenol composition among different dietary berries, we may expect to see differential effects in modulating biomarkers of CVD risks. Nonetheless, all berry products have shown consistent evidence of lowering one or more biomarkers of conventional lipids in reported studies assessing lipid outcomes. 

## 3. Conclusions

Reported studies have shown a consistent role of dietary whole berries, as well as berry juices and extracts, in decreasing blood total and LDL cholesterol and triglycerides, and/or increasing HDL-C in subjects with one or more elevated lipid biomarkers. A few studies have further shown that dietary supplementation of freeze-dried whole berries can also improve qualitative changes in lipids, such as by decreasing small HDL and LDL particle concentrations and increasing LDL size, thus conferring less atherogenicity and reducing CVD risks. These clinical observations have been explained by mechanistic studies demonstrating the role of dietary berries in modulating lipid metabolism, mainly by increasing hepatic synthesis of apolipoprotein A-I, downregulating the activity of genes related to fatty acid synthesis, causing regression of aortic lesions, and decreasing inflammation and oxidative damage in experimental animals. In addition to lowering lipids, clinical studies have also provided evidence on the role of dietary berries in decreasing surrogate markers of atherosclerosis, including biomarkers of oxidative stress and inflammation. Most reported studies have revealed the effects of berries in modulating oxidized lipids and nitric oxide; only one study showed a decrease in CRP, while others revealed no changes in biomarkers of inflammation. However, there is a large heterogeneity in the biomarkers reported by each study, and thus, further investigation is needed on the effects of berries on markers of atherosclerosis and endothelial function in clinical settings. Additionally, most of the reported studies consisted of a small sample size and involved a short duration of intervention, which must be addressed in future trials. Finally, sources of funding can play an important role in the interpretation of reported results. Thus, we reviewed the funding sources of the clinical trials included in our communication and it appears that most trials were funded by a combination of nonprofit and for profit sources, which may minimize related bias. However, in most cases, the test agents have been supplied by specific commercial food or nutraceutical companies, and this must be taken into consideration when comparing and contrasting results among the different trials.

## 4. Future Research and Recommendations

Based on the emerging evidence, dietary berries and berry products hold promise as a natural and alternative means to lower blood lipids in adults with CVD risks. On the basis of epidemiological and clinical findings, including two cups of low calorie cranberry juice or half to one cup of whole berries in the daily diet may provide these health benefits in adults. However, it is important that these recommendations must be made in the context of the existing diet and the magnitude of the CVD risks present in individuals. Most of the studies reviewed herein were in overweight/obese but otherwise healthy adults, and thus, recommendations might differ for those with advanced CVD and type 2 diabetes. Additionally, some of the clinical studies used a large dose of berries not feasible to consume on a daily basis. Thus, future studies must address the dose-response effects of berry products in modulating lipids and related cardio metabolic variables with or without changes in background diet and lifestyle. Studies comparing the effects of berry products with conventional lipid-lowering medications, especially targeting LDL-C, will be useful in assessing the magnitude of absolute risk reduction of CVD among different therapies. 

In addition to the determination of blood lipids and lipoproteins, future studies must also examine the effects of berry supplementation on the changes in the gut microbiome that have been significantly associated with risks of chronic diseases, including blood lipids [50,51]. Inter-individual variations in diet, lifestyle factors, and gut microbiome may further explain and modify lipid and metabolic responses to dietary berry interventions, and such studies will help identify personalized approaches for optimal lipid management in lowering CVD risks. 

## Figures and Tables

**Table 1:** Effects of dietary berries (whole fruit, juice, or freeze-dried berries) on serum lipids: clinical studies.

Author (Year) Funding	Study Design	Participants	Intervention	Significant Effects on Conventional Lipids	Significant Effects on Lipid Subclasses/Apolipoproteins
Ruel et al. [35] Canadian Cranberry Growers Coalition	Four week successive periods of intervention with increasing doses of CJC	Obese men (*n* = 30)	Three doses of CJC (125, 250, and 500 mL) vs. placebo juice/day	Increases in plasma HDL cholesterol (46 ± 5 to 49 ± 6 mg/dL) and decreases in ratio of total and HDL cholesterol with increasing doses of CJC vs. placebo	No significant effects on lipids
Burton-Freeman et al. [41] California Strawberry Commission	Randomized crossover trial; 12 wks	Hyperlipidemic adults (*n* = 24)	Strawberry beverage (10 g FDS) vs. matched placebo with or without high-fat meal challenge	Decrease in postprandial triglycerides in the strawberry (131 ± 2 mg/dL) vs. placebo (13 ± 2 mg/dL) group	Not reported
Zunino et al. [34] USDA, California Strawberry Commission	Randomized cross-over trial; 7 wks	Obese adults (*n* = 20)	Strawberry beverage (4 servings strawberries) vs. strawberry-flavored control beverage	Decreases in total cholesterol (182 ± 38 to 169 ± 37 mg/dL) in the strawberry vs. control group	Decreases in NMR-derived small HDL particle concentrations (18.3 ± 4.4 to 17.2 ± 3.8 µmol/L), and increase in LDL size (21 ± 0.7 to 21.22 ± 0.6 nm) in the strawberry vs. control group
Basu et al. [13] NIH NCRR, California Strawberry Commission	Randomized parallel trial; 12 wks	Adults with above optimal serum lipids (*n* = 60)	High-dose FDS (50 g/d), low-dose FDS (25 g/d) vs. fiber and calorie-matched control beverages	Decreases in total (214 ± 7 to 181 ± 5 mg/dL) and LDL-C (130 ± 7 to 103 ± 5 mg/dL) in high-dose vs. low-dose FDS and controls at 12 wks vs. baseline	Decreases in NMR-derived small LDL particle concentrations (697 ± 106 to 396 ± 69 nmol/L) in high-dose vs. low-dose FDS and controls at 12 wks vs. baseline
Lankinen et al. [42] funders including non-profit and for profit entities in Finland	Randomized parallel trial; 12 wks	Overweight/obese adults with metabolic syndrome (*n* = 131)	Healthy diet (whole grains, fish, bilberries (300 g/day), whole grain diet (whole grains), or control diet (refined grains)	Increase in large HDL particle concentrations and particle size in the healthy diet group vs. control	No changes in Apo-A-I and Apo-B 100 between diets
Novotny et al. [36] USDA, Ocean Spray Cranberries, Inc.	Randomized parallel trial; 8 wks	Overweight adults (*n* = 56)	LCCJ or matched placebo beverage (480 mL)/day	Decreases in serum triglycerides (113 ± 9 to 102 ± 4 mg/dL) in the LCCJ vs. placebo at 8 wks	No changes in Apo-A-I, A-II, or Apo-B

Apo: apolipoprotein; CJC: cranberry juice cocktail; FDS: freeze-dried strawberries; LCCJ: low-calorie cranberry juice; NCRR: National Center for Research Resources; NIH: National Institutes of Health; NMR: nuclear magnetic resonance; USDA: United States Department of Agriculture.

**Table 2:** Effects of berry bioactive compound extracts on serum lipids: clinical studies.

Author (Year) Funding	Study Design	Participants	Intervention	Significant Effects on Conventional Lipids	Significant Effects on Lipid Subclasses/Apo Lipoproteins
Lee et al. [30] Academic grant from Taichung, Taiwan	Randomized parallel trial; 12 wks	Adults with type 2 diabetes (*n* = 30)	Cranberry extracts (3 capsules/day ≈ 1500 mg extracts) vs. placebo	Decreases in total (−16 ± 4 mg/d/L) and LDL cholesterol (−15 ± 4 mg/dL) in the cranberry vs. placebo	Not reported
Qin et al. [26] National Natural Science Foundation of China	Randomized parallel trial; 12 wks	Adults with dyslipidemia (*n* = 120)	Anthocyanin extracts (4 capsules/day ≈ 320 mg extracts) vs. placebo	Decreases in LDL (159 ± 34 to 140 ± 35 mg/dL) and increases in HDL cholesterol (46 ± 8 to 51 ± 9 mg/dL) in anthocyanin vs. placebo	Decreases in CETP mass and activity in anthocyanin vs. placebo; no changes in Apo-A-I and Apo-B
Broncel et al. [31] University of Łódź, Poland	Uncontrolled study; 2 months vs. baseline	Healthy adults (*n* = 22) and adults with MS (*n* = 25)	Chokeberry extracts (300 mg/day)	Decreases in total (243 ± 35 to 228 ± 33 mg/dL), LDL cholesterol (159 ± 36 to 146 ± 35 mg/dL), and triglycerides (216 ± 67 to 188 ± 90 mg/dL) in berry group vs. baseline	Not reported
Zhu et al. [27] National Natural Science Foundation of China	Randomized parallel trial; 12 wks	Adults with hypercholesterolemia (*n* = 150)	Anthocyanin extracts (4 capsules/day ≈ 320 mg extracts) vs. placebo	Decreases in LDL (130 ± 22 to 117 ± 16 mg/dL) and increases in HDL cholesterol (47 ± 9 to 53 ± 8 mg/dL) in anthocyanin vs. placebo	No changes in Apo-A-I and Apo-B
Soltani et al. [32] No funding source reported	Randomized parallel trial; 4 wks	Adults with hyperlipidemia (*n* = 50)	Whortleberry extracts (90 mg anthocyanins) vs. placebo	Decreases in total (225 ± 32 to 192 ± 29 mg/dL), LDL cholesterol (133 ± 24 to 122 ± 27 mg/dL) and triglycerides (226 ± 97 to 156 ± 47 mg/dL) in the berry group vs. placebo	Not reported
Kianbakht et al. [33] Research Institute for Islamic and Complementary Medicine	Randomized parallel trial; 8 wks	Adults with hyperlipidemia (*n* = 80)	Whortleberry extracts (1050 mg fruit extracts) vs. placebo	Decreases in total (282 ± 38 to 202 ± 37 mg/dL), LDL cholesterol (172 ± 48 to 117 ± 35 mg/dL) and triglycerides (305 ± 23 to 248 ± 19 mg/dL), and increases in HDL cholesterol (44 ± 5 to 59 ± 7 mg/dL) in the berry group vs. placebo	Not reported

Apo: apolipoprotein; CETP: cholesteryl ester transfer protein; MS: metabolic syndrome.

**Table 3:** Effects of dietary berries and markers of atherosclerosis and inflammation: clinical studies.

Author (Year) Funding	Study Design	Participants	Intervention	Significant Effects on Surrogate Markers of Atherosclerosis	Significant Effects on Biomarkers of Inflammation
Basu et al. [12] NIH NCRR, California Strawberry Commission	Randomized parallel trial; 8 wks	Adults with MS (*n* = 27)	FDS (50 g/day) vs. control beverage	Decreases in VCAM-1	Not reported
Basu et al. [12] NIH NCRR, US Highbush Blueberry Council	Randomized parallel trial; 8 wks	Adults with MS (*n* = 48)	FDB (50 g/day) vs. control beverage	Decreases in plasma oxidized LDL and MDA; no effects on adhesion molecules	No effects on CRP and IL-6
Basu et al. [44] Cranberry Institute and Ocean Spray Cranberries, Inc.	Randomized parallel trial; 8 wks	Adults with MS (*n* = 31)	LCCJ (480 mL/day) vs. matched placebo	Decreases in plasma oxidized LDL and MDA	No effects on CRP and IL-6
Simao et al. [48] National Council of Brazilian Research	Randomized parallel trial; 60 d	Adults with MS (*n* = 56)	Cranberry juice vs. no juice (usual diet as control group)	Decrease in homocysteine, hydroperoxides and AOPP	Increase in adiponectin; no change in CRP, IL-1,6, and TNF-α
Novotny et al. [36] USDA, Ocean Spray Cranberries, Inc.	Randomized parallel trial; 8 wks	Overweight adults (*n* = 56)	LCCJ or matched placebo beverage (480 mL)/day	No changes in adhesion molecules	Decrease in CRP
Johnson et al. [49] USDA, US Highbush Blueberry Council	Randomized parallel trial; 8 wks	Postmenopausal women with hypertension (*n* = 48)	FDB (22 g/day) vs. control powder	Increase in nitric oxide	No effects on CRP

AOPP: Advanced oxidation; CRP: C-reactive protein; FDB: freeze-dried blueberries; FDS: freeze-dried strawberries; protein products; IL-6: interleukin-6; LCCJ: low-calorie cranberry juice; MDA: malondialdehyde; MS: metabolic syndrome; NCRR: National Center for Research Resources; NIH: National Institutes of Health; TNF-α: tumor necrosis factor-alpha; USDA: United States Department of Agriculture; VCAM-1: vascular cell adhesion molecule-1.
